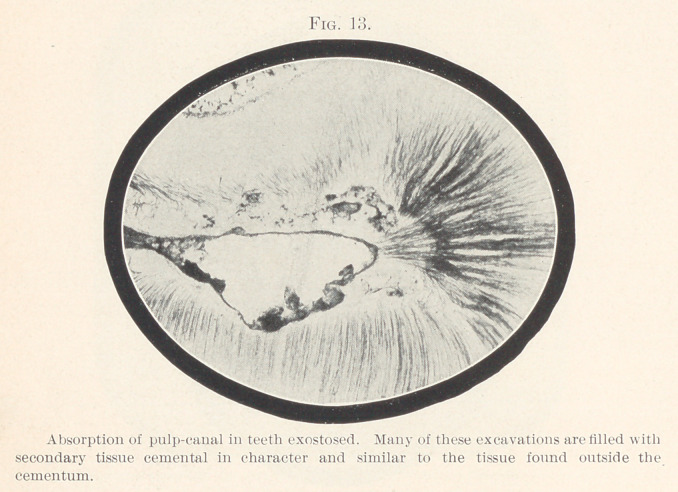# The Development of Hard Tissue in the Pulp of Human Teeth

**Published:** 1904-04

**Authors:** Douglas E. Caush

**Affiliations:** England


### THE DEVELOPMENT OF HARD TISSUE IN THE PULP OF HUMAN TEETH.

BY DOUGLAS E. CAUSH, L.D.S., ENGLAND.

Of the functions performed by the various organs of the body there are few, if any, more interesting or have the power of producing more varied results than that of the tooth-pulp.

In health it is the benefactor of the surrounding hard tissue; in disease, the medium whereby we are warned of the mischief that is going on. Among the varied functions of this organ may be placed the power of developing "secondary dentine."

Fig. 1.

*a*, pulp-nodule showing structureless character of secondary dentine.

Nature in many cases endeavors to overcome the evil produced by the destroying microbe, by forming a layer of fresh tissue between the soft, sensitive pulp and the dentine that is being broken down by decay. The same protective measure also occurs in those cases where erosion has commenced its destructive work.

as well as in many other instances where disintegration of the hard tissue has been produced by the various known causes. In all these examples the newly formed hard tissue is known as secondary dentine, the development of which we desire to consider in this paper.

On the examination of teeth, we find we may have new tissue more or less continuously developed from the pulp, until the tooth is lost either by disease or as a result of old age; in other cases the new tissue may be developed without any apparent cause, and the development may be either continuous or spasmodic, until the whole of the pulp-chamber becomes filled with this new tissue.

Fig. 2.

*a*, osteodentine.

Again we may find it in the pulp itself more or less surrounded by pulp-tissue in the form of pulp-nodules, and as Professor Jos. Arkövy has designated certain growths, "Odontoma interum liberum."

In any and all of these cases the new tissue is spoken of as secondary dentine; it will thus be seen that there is a variety of tissue grouped under the head of secondary dentine, ranging from the comparatively structureless tissue as seen in the pulp-nodule (Fig. 1) to that of the complicated character known as osteodentine (Fig. 2).

It may enable us to better understand the development of this tissue if we divide it into classes. We shall therefore consider, first, normal secondary dentine; second, dentine of repair; third, pulp-nodules; and lastly, a form of secondary dentine found in teeth where pyorrhœa alveolaris has been pronounced.

In the first class we shall include all tissue produced apparently without any cause, the whole of the pulp and surrounding tissue being healthy at the time of the deposition of the tissue; a good example of such may be frequently found in those teeth perfectly free from diseases such as decay or erosion, nor have the teeth been worn down by attrition, yet on examination they are found to have their pulp-chambers partially or entirely filled with new tissue. This tissue may be very varied in its microscopic appearance, from that of an almost structureless character to the more complicated, containing lacunæ-like spaces and frequently an innumerable number of tubuli (Fig. 3); the tissue is usually

Fig. 3.

Pulp-chamber superior bicuspid, formation of secondary dentine.

found in the twelve anterior teeth, but perhaps more frequently in the lower incisors and canines; there is, as a rule, no inconvenience produced by its development, and its presence is only detected on examining the tooth after extraction.

It would appear to be Nature's way of reducing the size of the pulp-chamber, and is much more pronounced in those who have passed age. It is rarely found in the pulp-canals; the deposition commences usually at or near the apex of the pulp-chamber, gradually working downward towards the point where the chamber is united to the pulp-canal; even when the tubuli are in large numbers they rarely assimilate with those tubuli previously formed, hence it is not at all difficult to define the line of demarcation between the two tissues. This line is usually very pronounced,

Fig. 4.

Secondary dentine under decay: *a*, decay; *b*, secondary dentine.

owing to the fact that some time has elapsed between the calcification of the old tissue and the commencement of the deposition of the new, and, as a consequence, the first layer laid down usually appears structureless and often followed by a number of irregular spaces very similar in character to the inter-granular layer of the dentine; and then, if the deposition is continued, tubuli are regularly formed. If, on the other hand, from any cause the deposition is interrupted, we have a mass of mixed tissue, structureless, lacunæ-like spaces, and tubuli, all mixed together.

The development of this tissue is as follows: After the dentine has become perfectly calcified in a healthy tooth, fresh formative material is brought to the pulp by the blood; this, passing to the outer layer of the pulp by capillaries, is taken up by the layer of globular or semi-globular cells surrounding the odontoblasts (Figs. 4, 5, 6, 7, 8), and these cells become eventually calcified. As this calcification takes place the odontoblasts are restricted in size by compression and the odontoblast cells forced inward towards the

Fig. 5.

Section of superior canine worn away at the sides by a metal band on artificial tooth plate; though the tooth is worn away by attrition, the deposition assimilates that found in eroded teeth: *a*, portion of tooth worn away by band; *b*, deposition of secondary dentine.

centre of the pulp, the constricted portion forming the tubuli; if the deposition is slow, the outer mass becomes very solid, and, as a consequence, structureless with few tubuli; if, on the other hand, it is rapid, some of the cells do not calcify, and lacunæ-like spaces are thus formed with their canaliculi produced as minute spaces between the calcified cells. As this process goes on new cells are formed by cell division. If during the process of calcification any dense tissue is met, such as the walls of the blood-vessels (arteries or veins), the cells surround this dense tissue, and, calcifying around them, form the circular openings so frequently seen in the sections of this tissue, often carrying by pressure the odontoblasts with them, and thus tubuli are seen surrounding the openings; if, on the other hand, no dense substance is met, the calcification goes on continuously until the whole of the pulp-chamber is filled with new tissue, thus producing the variations found in this tissue.

The cause of all this may be—probably is—the slight irritation —" so slight that the patient is unconscious of it"—produced in the pulp by the constant use of the teeth.

Fig. 6.

Pulp-nodule surrounded by hard and soft tissue: *a*, pulp-nodule; *b*, soft tissue; *c*, hard tissue. For nodule surrounded by soft tissue, see Fig. 1.

The second class, and by far the most numerous, spoken of as dentine of repair, may be divided as follows, the pulp-nodule being produced from quite a different cause,—" irritation produced in the pulp itself:" 1, that found in teeth with eroded surfaces; 2, found in teeth that are decayed; 3, found in teeth worn down by attrition and mastication.

On the microscopic examination of the teeth placed in Section 1, " teeth with eroded surfaces" (Fig. 9), we have found in every tooth examined that there has been a deposition of secondary dentine. This new tissue assimilates that of normal dentine more than in those of the other sections; the line of demarcation is more pronounced following the area of the eroded surface; it is much more clearly defined, and certainly more restricted in its development, having, as a rule, tubuli assimilating with the tubuli of the dentine, and apparently there is little or no break in their continuity; at the same time there appears to have been a marked

Fig. 7.

Pulp-nodule attached to original dentine with tubuli curved around nodule.

change in the condition of the tubuli of the original dentine. Immediately under the eroded surface we find the tubuli of the original dentine very difficult, if not impossible, to stain; it appears as if these tubuli had been filled with some deposit from the outer surface of the tooth that prevents them from performing their usual functions. The function of the secondary dentine is to prevent any acute irritation passing from the eroded surface to the pulp itself; owing to the restricted area of its development, it would appear as if the deposition was caused by the slight local irritation produced in the earlier stages of the disease of erosion.

The development of secondary dentine in the second section is, so far as my examination goes, much more rare, and is found only in those cases where the decay has gone on slowly (Fig. 10) and the irritation to the pulp-tissue has been as a consequence slight and continuous but extending over a long period, differing from the former in microscopic structure. It varies very much in

Fig. 8.

Nodule at apex of root-canal, in this position causes great pain. The dense tissue almost surrounding nodule is pulp.

structure as well as in the area of its deposition. The earlier deposited tissue does not, as a rule, contain any tubuli, but a number of lacunæ with canaliculi; as the tissue increases in thickness it assumes more the character of normal dentine, and a number of tubuli are developed; it usually spreads over a much larger area than that of the secondary dentine in eroded teeth, and does not terminate in such a pronounced or irregular manner. It would lead one to suppose that the irritation to the pulp-tissue had been much more general; it is still an attempt by Nature to protect the pulp from the results of the diseased condition of the tooth produced by the action of the spreading of the decay. In the form of secondary dentine, placed in the third division, that produced by attrition or mastication (Figs. 11 and 12), we have the teeth worn down oftentimes in extreme cases until the original pulp-chamber has become exposed. The teeth usually affected by attrition are the six front ones of both upper and lower jaw; those worn down by mastication, the bicuspids and molars. In either case

Fig. 9.

Section of pulp-nodule taken from pulp-chamber of superior molar.

the result is the same. The tissue is usually quite structureless at the commencement of the deposition, passing from that through either of the other forms until we have a mass of dense tissue all fused together and entirely filling the pulp-chamber, so dense that, in extreme cases, even if the original pulp-chamber has been exposed, there is little or no discomfort to the patient. This tissue is more like the tissue previously referred to as normal secondary dentine than that of any of the others. In all these cases the method of development has been the same, and the cause that of irritation in one form or another, but in *all* cases the exciting cause has been from the outside,—*i.e.,* from that portion of the tooth above the gum line.

In the development of the pulp-nodule we have a pronounced deposit of secondary dentine either in the pulp-chamber or the pulp-canals, sometimes in both, but, unlike the deposit in the previous sections, the nodule is always formed as a result of irritation *in the pulp itself.*

Fig. 10.

Pulp-nodule showing what I believe to be the origin of nodule at *a*.

On examining a tooth there is nothing in the external appearance to suggest the existence of even the smallest of pulp-stones, nor can we take into consideration as a factor the age or sex of patients in the development of this tissue, as these nodules are to be found in the teeth of patients in their teens as well as in those of advanced age, and in many cases producing as much pain in the youngest as in those of old age. The result of my examinations tends to show that the position of the nodule is usually the cause of the pain produced.

In external appearance the nodules vary from a small, more or less globular, or oval, structure to that of any size or shape, controlled only by the size of that portion of the canal or chamber in which they are found. Thus we have them from a minute point to those entirely filling the pulp-chamber, and in teeth of two or more roots it is not unusual to find them not only filling the pulp-chamber, but with spines of hard tissue passing into the various canals, and thus producing the most irregular-shaped nodules.

Fig. 11.

Interglobular spaces in dentine as seen in teeth affected by pyorrhœa alveolaris. Note the globular character of the dentine.

Their position is almost as varied as their outline, for, as I have already said, we may find them in the pulp-canals and pulp-chambers, quite free from the surrounding dentine (Fig. 13), or we may find them attached to the sides of the pulp-canals and quite surrounded by the dentine. It is not the largest of these that cause the greatest amount of pain, as the pain is produced by the position, and not by the size of the nodule; thus a small nodule near the apex of the canal will probably produce pain by the constriction of the pulp and, as a consequence, pressure upon the nerves, whilst a nodule much larger in size (unless there is pressure produced upon the nerves) may almost entirely fill the pulp-chamber without producing any discomfort. Though their size, shape, and position may vary very much, such is not the case with regard to their microscopic structure. All the nodules I have examined have a somewhat similar structure when viewed by the microscope. We have usually in the centre, or somewhere near the centre, of the developed tissue a space, more or less pronounced, and it is at this point that the nodule has its origin; it grows outwardly, and radiating from this point there are

Fig. 12.

Absorption of dentine in teeth having pyorrhœa alveolaris.

usually a number of more or less concentric rings caused by additional layers of calcified tissue; it is in this way a nodule increases in size. This may continue until two or more nodules touch each other and become united into one, thus forming a compound nodule large and irregular in shape. Its structure, as thus seen, is quite different from either ordinary or secondary dentine. We may sometimes find a few isolated irregular tubuli, but rarely do we find any tubuli approaching the character of those seen in secondary dentine. This may be accounted for by the fact that its origin is generally some distance from the odontoblastic layer, and the nodule is developed from a different layer of cells from that of secondary dentine. Its position would imply that no odontoblasts had taken part in its development, and, if this is so, a very interesting question arises as to how these tubuli, if they are tubuli, are produced.

The origin of the development of the nodule is some irritation *in the pulp itself,* and I believe the primary object of the nodule is to cover up, by calcification, some substance that has been the cause of irritation in the pulp-tissue. Its structure as well as its mode of development would lend itself to this supposition, for under the

FIG. 13.

Absorption of pulp-canal in teeth exostosed. Many of these excavations are filled with secondary tissue cemental in character and similar to the tissue found outside the cementum.

microscope we have a structure similar in appearance and, I believe, identical in its mode of development to that of the pearl found in the oyster, the origin of the pearl being a foreign body found in the mantle of the mollusc. As it is impossible for the oyster to get rid of this foreign body by absorption, it builds around the cause of irritation that which is known to us as the pearl. So in the development of the pulp-nodule the same has occurred. Nature has found in that delicate and complicated structure, the pulp, something it cannot get rid of, something it cannot absorb. The irritation produced by this something causes the pulp to endeavor to get rid of it; as, however, it cannot absorb it, there is nothing for it to do but to encyst it.

If this is the true origin of the pulp-nodule, we ought to be able to find in some pulps, as a result of microscopic examination, the exciting cause, and this exciting cause is, I believe, to be found in either a dead cell or cells, or perhaps a few blood-corpuscles that have by some means escaped from their ordinary course, by the rupture either of one of the capillaries or small blood-vessels abounding in the pulp; such an accident may occur by a sudden shock to the tooth, or, again, the corpuscles may be found out of their place as a result of the breaking down of some of the smaller arteries, veins, or even the capillaries, by disease such as the pulp is exposed to.

I think the former method is the one that usually causes the development of pulp-nodules in our younger patients, whilst undoubtedly the changes in the pulp lend themselves to the development of these nodules as age advances. We will suppose, from either of the above, or some similar cause, that such a change has taken place in the pulp, and as a result there will be irritation of a more or less pronounced character in this tissue produced by what has become a foreign body; there are no lymphatics for the reabsorption of this body, and it does not require a very great stretch of the imagination to trace the growth of the pulp-nodule on the lines laid down. The course followed would, I believe, be somewhat as follows:

The irritation produced by the foreign body causes an increased activity in the blood-vessels surrounding the cause of the irritation, and, as a necessary consequence, an increase of formative material brought to hand. This material is taken out of the blood by the surrounding cells, and it becomes a very simple matter for these cells to cover up the cause of the irritation by a deposition of hard tissue. This probably occurs as follows: the increased blood-supply, produced by the irritation, causes immediate activity in the cells surrounding the cause of irritation, and the result of the activity is to produce a number of new cells. The pressure produced by this increase in the number of cells again increases the blood-supply, and after a time the cells deposit a hard tissue in the same manner as the cementum is produced in exostosis.

As the deposition takes place in very small quantities, and probably very slowly, we have a more or less perfectly calcified and, as a consequence, homogeneous deposition. This accounts for the structureless character of the nodule.

Again, we sometimes have a larger mass more rapidly formed, and perhaps nearer, or even including some of the odontoblasts in the newly forming mass. We shall then have variations in the structure, consisting of lacunæ with canaliculi, irregular spaces, and a few markings like the tubuli of dentine. As the calcification of these cells continues, we shall have an increase in the size of the nodule; in some cases this increase causes a further irritation of the surrounding pulp-tissue, and thus a constant supply of fresh formative material is brought to the cells. The increase of size eventually produces pressure upon the nerves of the pulp and, as a consequence, frequently the most acute pain is experienced; this is the course, I believe, of the pulp-nodule when surrounded by pulp-tissue.

There is yet another class of nodule; though previously mentioned, it may be interesting to briefly follow its history. I refer to those nodules attached to the dentine. We may also, if we are fairly successful in our search, find them not only attached at one side, but entirely surrounded by dentine, and that at some little distance from the pulp-canal; wherever I have found these they have always been near the apex of the root and embedded in the last formed dentine; and this, I think, gives the key to the explanation of the position in which the nodules are found.

At the time the cutting edge of the tooth is passing through the gums the apex of the root is in an uncalcified condition, and with a new tooth in this position it is much more subject to a shock than it will be after it is fully erupted; as a consequence, we have a nodule formed in precisely the same manner as those formed in the pulp-tissue, with the exception that those found in the pulp-tissue are always formed after the calcification of the dentine has taken place, whilst the latter class are formed prior to the calcification of the tooth, and when first formed are surrounded by uncalcified dentine. Any careful examination of a tooth with nodules in this position will fully illustrate my meaning, as the tubuli of the dentine will be found to be bent around the nodule, proving the nodule must have been formed prior to the calcification of the dentine. If it had been otherwise, and the dentine had been absorbed to make a space for the nodule, we should then have found the tubuli ending abruptly at or near the margin of the nodule, but this is not the case.

This will also account for the nodules found in the pulp-canals but attached to the dentine, and here, I believe, the development has been the same as in nodules surrounded by dentine.

The last of the forms of secondary dentine we wish to draw your attention to is to be found in the pulp-chambers and pulp-canals of teeth having been removed owing to the pronounced action of "pyorrhœa alveolaris." In all the teeth examined (about one hundred) the original dentine has been of very poor structure, containing a large number of interglobular spaces, the dentine, generally, badly calcified, and more or less absorption of the cementum and sometimes the dentine at the apex of the root. In the pulp itself the first thing that attracts our attention is the absorption of a portion of the original dentine forming the pulp-chamber and canal, probably the result of acute inflammation prior to the development of the secondary dentine. The new tissue is very varied in character, and apparently consists of a mixture of tissue similar in structure to that found in the previous cases, with the addition of a very large number of isolated globular secondary dentine wherever there is a piece of pulp-tissue; so that the whole of the pulp-chamber canals are more or less filled with either secondary dentine, as previously described, or an innumerable number of these globular bodies, generally isolated but sometimes though very rarely found fused together. I have noticed similar excavations in the pulp-canals of teeth that are exostosed, but with this difference: the cavities in the latter case are frequently filled with secondary tissue like the additional layer of cemental tissue on the outside; in the case of pyorrhœa alveolaris, the excavations are not filled with any special tissue.

All the microscopic slides used for illustrating this paper are hard sections ground down, as I believe decalcification in any form is liable to alter the conditions of the tissue under examination.

## Figures and Tables

**Fig. 1. f1:**
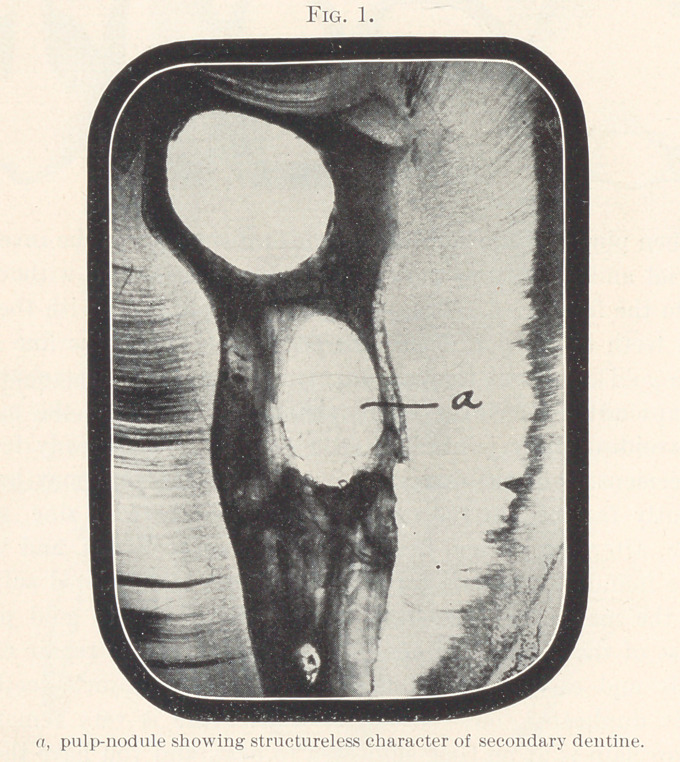


**Fig. 2. f2:**
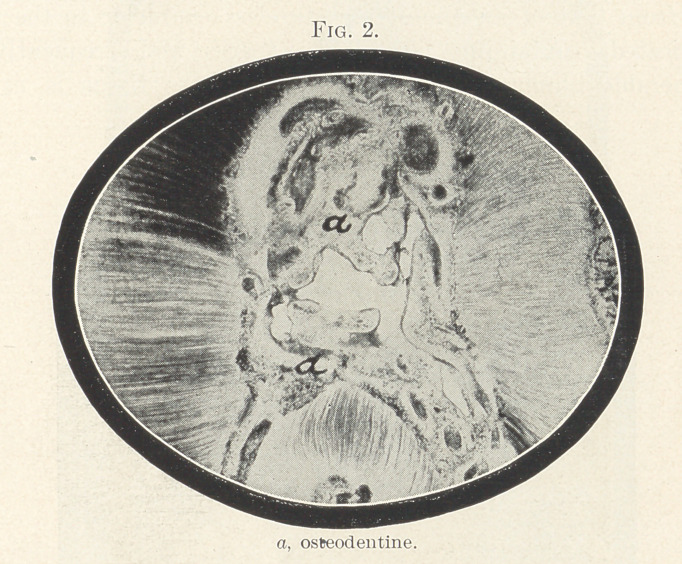


**Fig. 3. f3:**
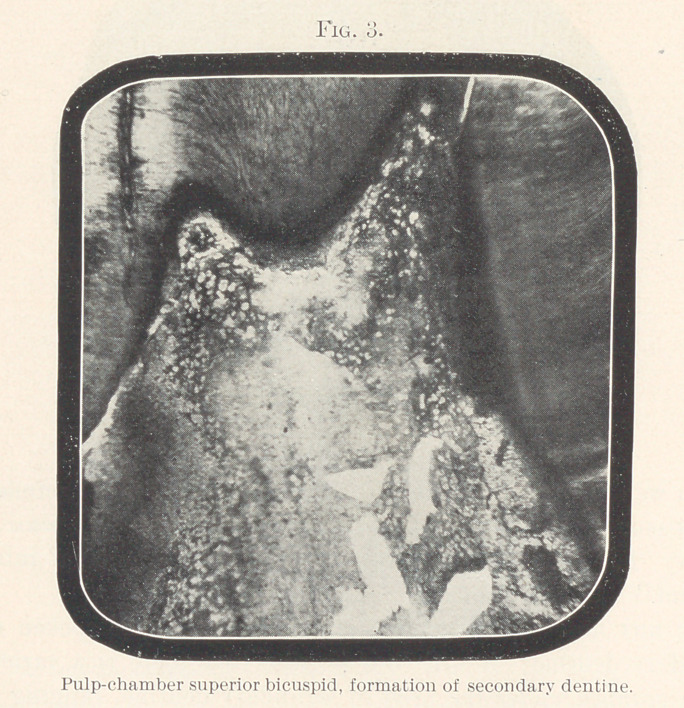


**Fig. 4. f4:**
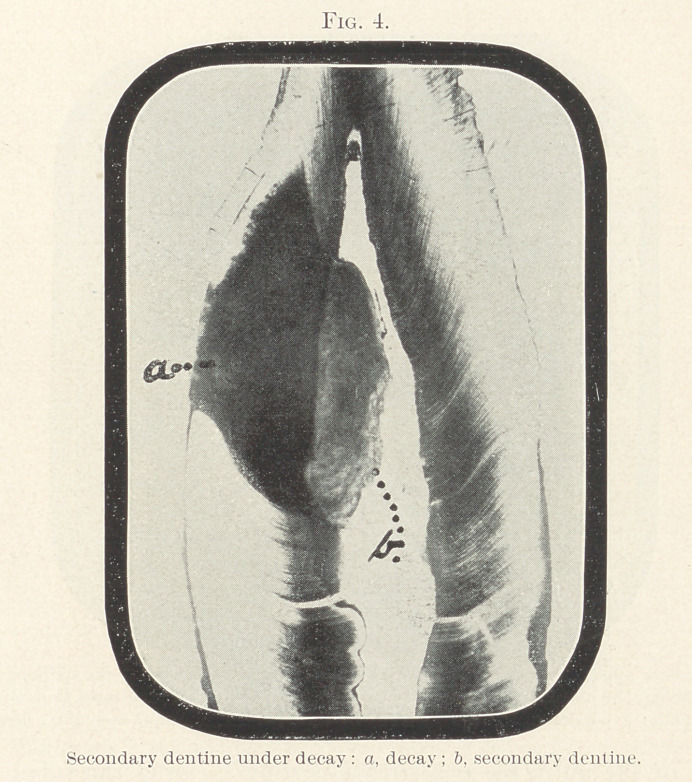


**Fig. 5. f5:**
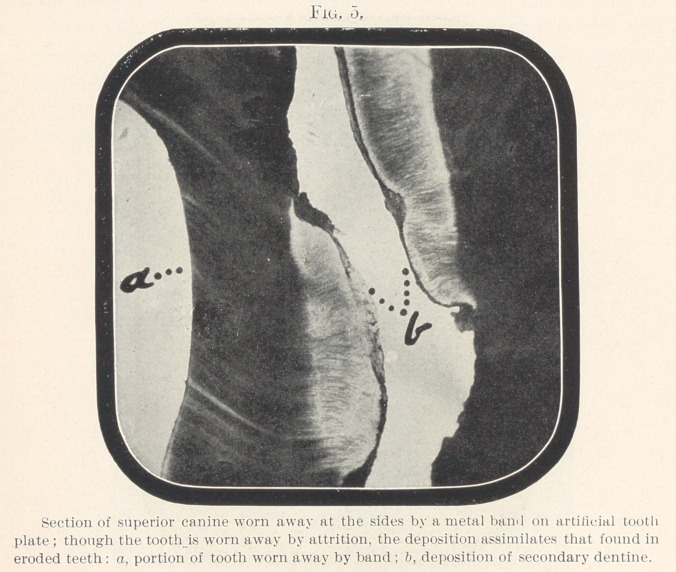


**Fig. 6. f6:**
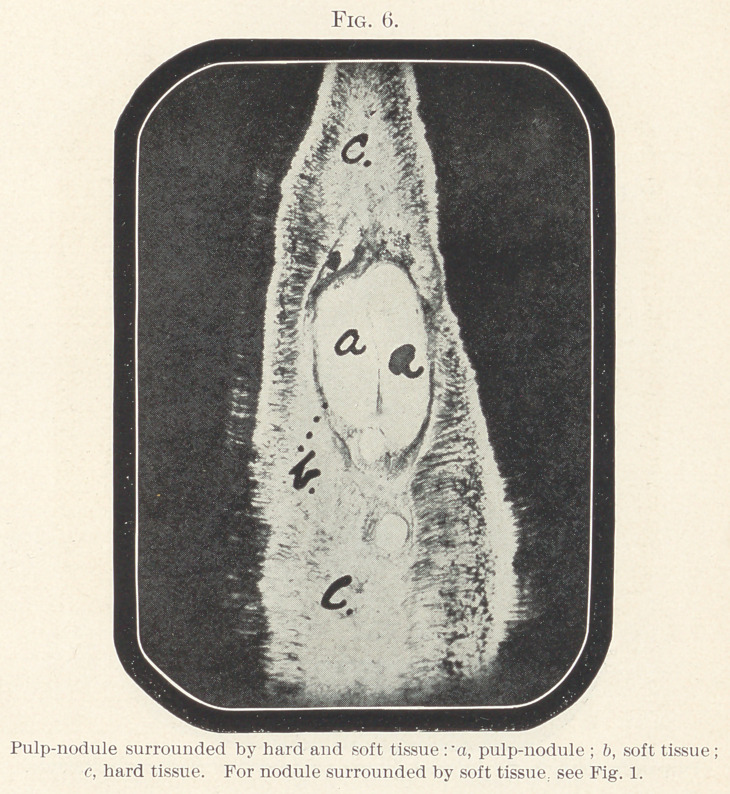


**Fig. 7. f7:**
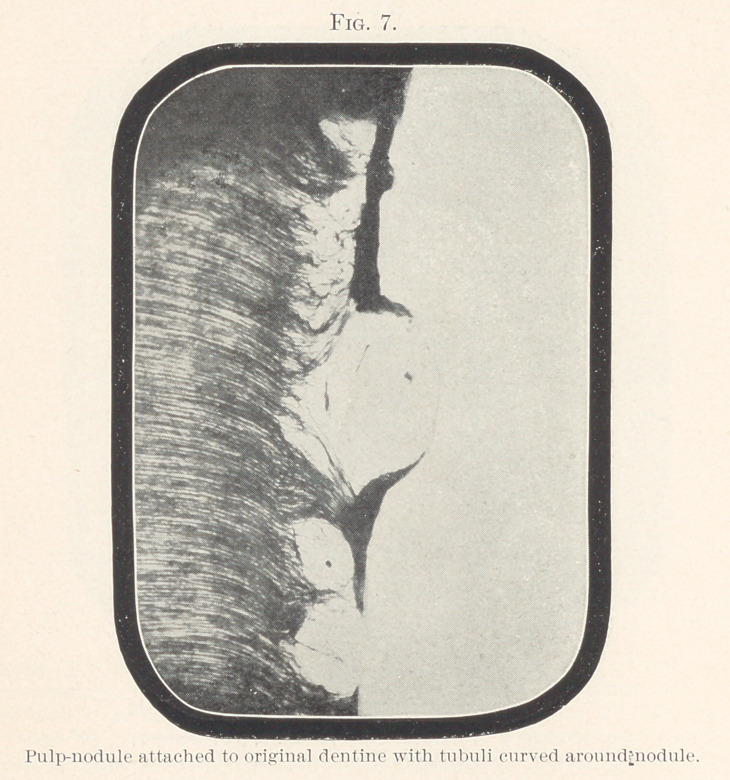


**Fig. 8. f8:**
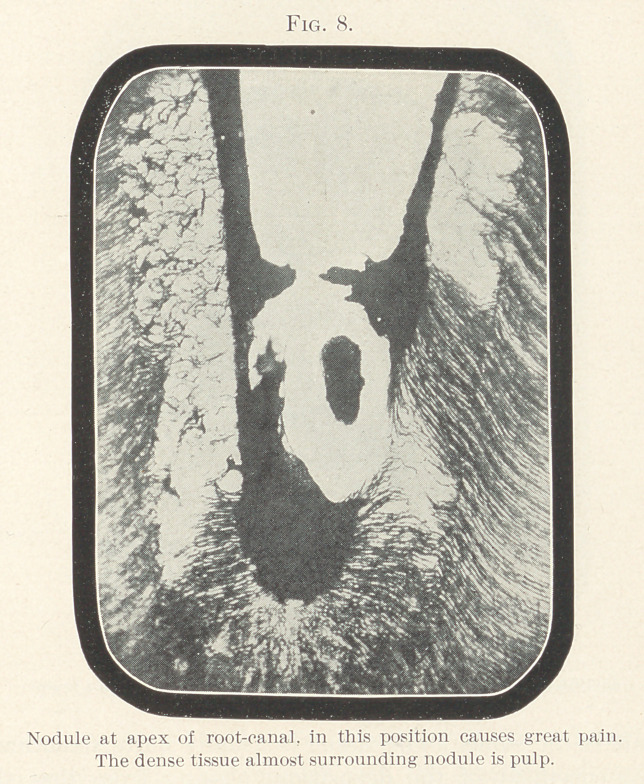


**Fig. 9. f9:**
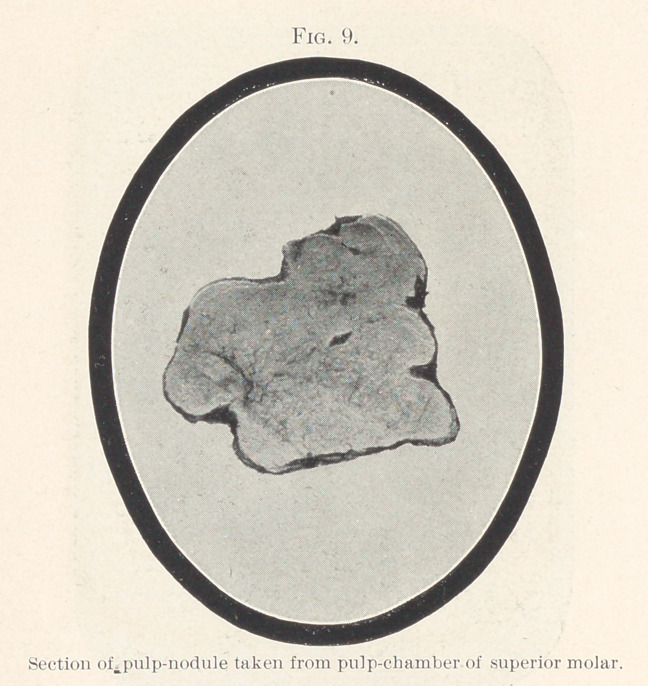


**Fig. 10. f10:**
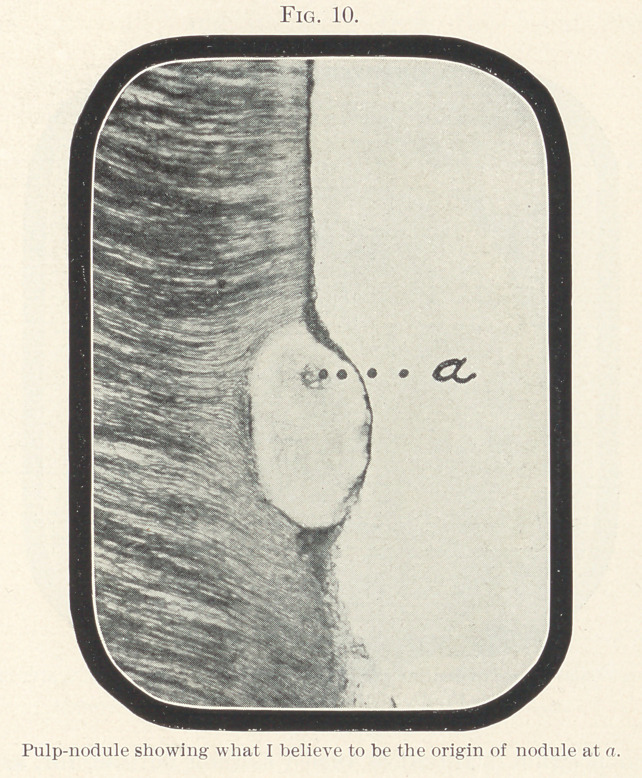


**Fig. 11. f11:**
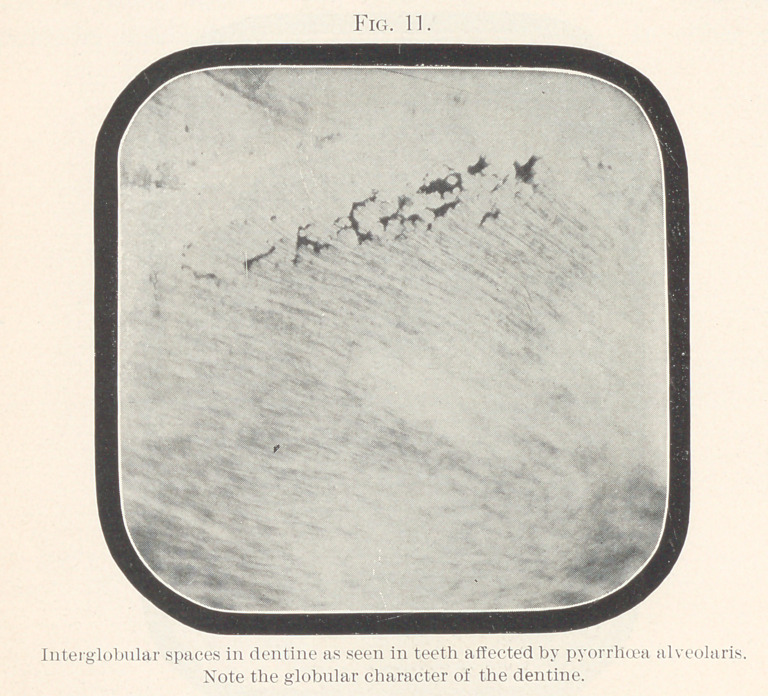


**Fig. 12. f12:**
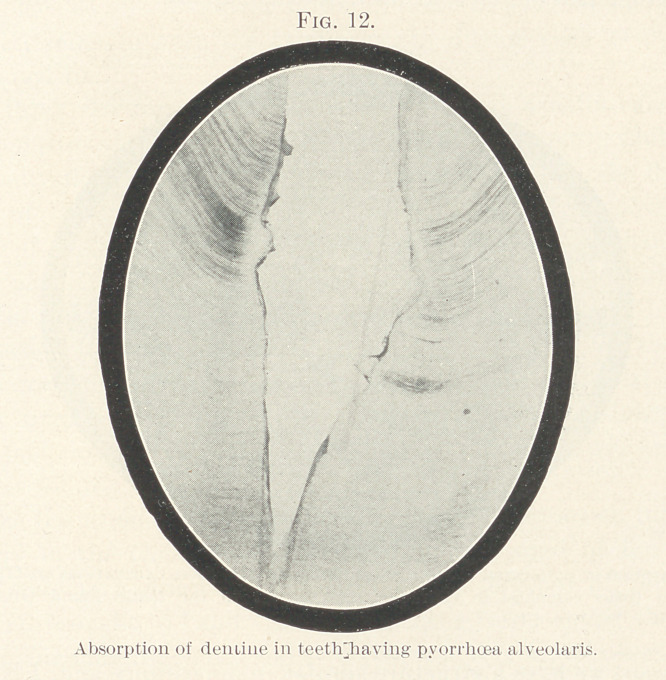


**Fig. 13. f13:**